# Camelid Single-Domain Antibodies (VHHs) against Crotoxin: A Basis for Developing Modular Building Blocks for the Enhancement of Treatment or Diagnosis of Crotalic Envenoming

**DOI:** 10.3390/toxins10040142

**Published:** 2018-03-29

**Authors:** Marcos B. Luiz, Soraya S. Pereira, Nidiane D. R. Prado, Naan R. Gonçalves, Anderson M. Kayano, Leandro S. Moreira-Dill, Juliana C. Sobrinho, Fernando B. Zanchi, André L. Fuly, Cleberson F. Fernandes, Juliana P. Zuliani, Andreimar M. Soares, Rodrigo G. Stabeli, Carla F. C. Fernandes

**Affiliations:** 1 Fundação Oswaldo Cruz, Fiocruz Rondônia, Porto Velho, 76812-245 Rondônia, Brazil; marcos.barros@ifro.edu.br (M.B.L.); soraya.santos@fiocruz.br (S.S.P.); nidi_reis@hotmail.com (N.D.R.P.); naancontato@hotmail.com (N.R.G.); akayano@gmail.com (A.M.K.); leandrosoaresmoreira@gmail.com (L.S.M.-D.); jusbbio@gmail.com (J.C.S.); fernando.zanchi@fiocruz.br (F.B.Z.); juliana.zuliani@fiocruz.br (J.P.Z.); 2 Instituto Federal de Educação, Ciência e Tecnologia de Rondônia, IFRO, Guajará-Mirim, 76850-000 Rondônia, Brazil; 3 Universidade Federal Fluminense, UFF, Rio de Janeiro, 24220-900 Rio de Janeiro, Brazil; andfuly@vm.uff.br; 4 Embrapa Agroindústria Tropical, Fortaleza, 60020-181 Ceará, Brazil; cleberson.fernandes@embrapa.br; 5 Universidade Federal de Rondônia, UNIR, Porto Velho, 76801-974 Rondônia, Brazil; 6 Centro Universitário São Lucas, UniSL, 76812-245 Porto Velho-RO, Brazil; 7 Plataforma Bi-institucional de Pesquisa em Medicina Translacional, Fiocruz-USP, Ribeirão Preto, 14049-900 São Paulo, Brazil; rodrigo.stabeli@fiocruz.br; 8 Departamento de Medicina da Universidade Federal de São Carlos, Demed-UFSCAR, São Carlos, 13565-905 São Paulo, Brazil

**Keywords:** crotoxin, CB, VHH, *Crotalus durissus terrificus*

## Abstract

Toxic effects triggered by crotalic envenoming are mainly related to crotoxin (CTX), composed of a phospholipase A_2_ (CB) and a subunit with no toxic activity (CA). Camelids produce immunoglobulins G devoid of light chains, in which the antigen recognition domain is called VHH. Given their unique characteristics, VHHs were selected using Phage Display against CTX from *Crotalus durissus terrificus*. After three rounds of biopanning, four sequence profiles for CB (KF498602, KF498603, KF498604, and KF498605) and one for CA (KF498606) were revealed. All clones presented the VHH hallmark in FR2 and a long CDR3, with the exception of KF498606. After expressing pET22b-VHHs in *E. coli*, approximately 2 to 6 mg of protein per liter of culture were obtained. When tested for cross-reactivity, VHHs presented specificity for the *Crotalus* genus and were capable of recognizing CB through Western blot. KF498602 and KF498604 showed thermostability, and displayed affinity constants for CTX in the micro or nanomolar range. They inhibited *in vitro* CTX PLA_2_ activity, and CB cytotoxicity. Furthermore, KF498604 inhibited the CTX-induced myotoxicity in mice by 78.8%. Molecular docking revealed that KF498604 interacts with the CA–CB interface of CTX, seeming to block substrate access. Selected VHHs may be alternatives for the crotalic envenoming treatment.

## 1. Introduction

Recently, the World Health Organization (WHO) reinstated snakebite envenoming into its Neglected Tropical Diseases portfolio [1]. This classification may help to intensify the fight against this public health problem, especially in Africa, Asia, Oceania and Latin America, where about 20,000–90,000 people die from snakebites, from an estimated 421,000–1,841,000 cases, every year [2]. In Brazil, during 2001–2012, about 27,200 snakebite cases and 115 related deaths were reported per year [3]. The *Bothrops* genus was responsible for most of the reported cases, followed by *Crotalus*, *Lachesis*, and *Micrurus* genera [3].

Although there is a lower incidence of crotalic envenomation, when compared to bothropic snakebites, these accidents present the highest lethality rate (1.87%) [4,5]. Envenoming is characterized by neurotoxicity and coagulation disturbances, along with muscle and renal toxicity [6]. Mild local injuries, mainly edema and erythema, may be observed [7,8]. Most envenoming symptoms are caused by the venom’s protein compounds [6]. About 90% of *C. durissus terrificus* venom is composed of proteins; among them are phospholipases A_2_ (PLA_2_) [9]. At 24 kDa, CTX, which constitutes about 50% of the venom’s protein content, is a heterodimer consisting of an enzymatically active PLA_2_ subunit (CB) and an acidic subunit with no enzymatic or toxic activity (CA) [10]. CTX is responsible for the envenoming’s neurotoxic, systemic myotoxic and nephrotoxic effects.

Treatment of envenoming is performed by administration of immunoglobulin G (IgG) or F(ab′)/F(ab′)_2_ fragments obtained mainly from hyperimmunized animals [11]. Despite different pharmacokinetic profiles of whole IgG and antibody fragments, no substantial differences in neutralizing toxic activity of some venoms could be noted [11,12]. Antivenom therapeutic safety can be compromised by failures in the product purification process [11,13]. Moreover, industrial production of serotherapics is unsatisfactory. Deficitary availability and accessibility, high product cost, horse use inviability in zoonose endemic countries, and a low number of laboratories involved in production are issues that need to be circumvented to improve antivenom production [11,14,15,16]. Thus, the search for alternative and complementary methods for snakebite treatments is constant [17,18].

Monoclonal antibodies (mAbs, 150 kDa), proposed as therapeutic agents [18,19,20,21], present a distribution similar to that of first-generation serotherapeutic agents (polyclonal IgGs) [12,22]. Besides that, mAbs require humanization [23], and should necessitate an oligoclonal preparation against major venom proteins [18]. In addition to mAbs, recombinant F(ab′) and scFv (single chain variable fragment) forms can be obtained [24]. With approximately 25 kDa, scFv are less immunogenic and present greater biodistribution, when compared to whole IgGs [25,26,27]. These fragments can be produced in prokaryotic expression systems [24,28]; however, they have high renal clearance [29]. Thus, multivalent forms, such as sc(Fv)_2_ (~60 kDa) and [sc(Fv)_2_] (~120 kDa) have been proposed [30]. The low stability, solubility and affinity of these molecules continue to be a challenge for their clinical application [31,32].

In addition to conventional IgG, camelids produce functional antibodies devoid of light chains and CH1 domains, referred to as camelid heavy-chain antibodies (HCAbs) with approximately 90 kDa [33]. The antigen recognition site of HCAbs is formed by a single domain called VHH [34]. With a size measured in nanometers and a small molecular weight (15 kDa), the VHH repertoire can be vector recombined, cloned and expressed, using a prokaryotic system in soluble form, resulting in fragments with high antigenic specificity and affinity [35]. A high identity with human VH (more than 80%) justifies VHHs’ low immunogenicity [36]. Longer CDRs, mainly CDR3, allow for the recognition of enzymatic sites by VHH, inaccessible to murine or human antibodies [37]. Furthermore, when compared to other antibody fragments, VHHs exhibit higher thermal stability [38].

In analyzing camelid VHH characteristics, along with the need to develop more effective antivenoms, this study aimed to select and characterize *Lama glama* VHHs able to inhibit the phospholipase, cytotoxic and myotoxic activities of CTX isolated from *C. durissus terrificus* venom.

## 2. Results

### 2.1. L. glama Immunization, Library Construction and Selection of Anti-Crotoxin VHHs

**Figure 1:**
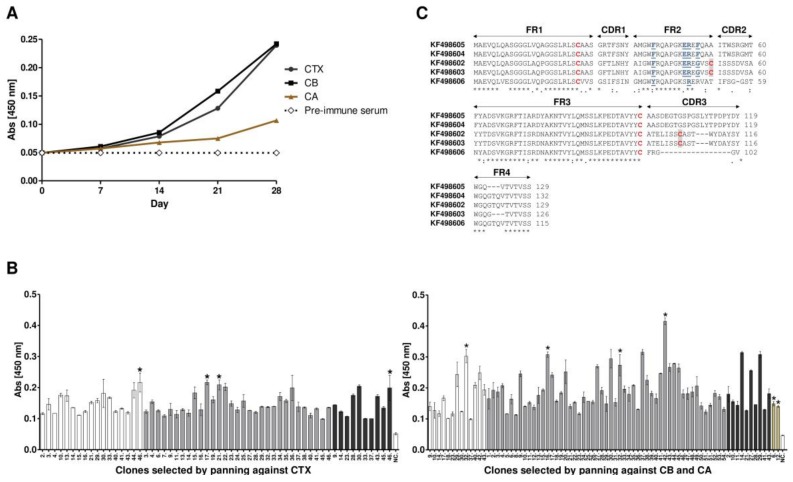
Development of anti-crotoxin VHH. (**A**) monitoring of the llama immune response. The serum was diluted to 1:10^6^ (*v*/*v*) and specific binding to CTX, CB and CA was detected by indirect ELISA. The animal showed a rapid and strong response after the second immunization (i.e., by day 14). Final boost performed on day 28 showed maximum absorbance, up to five times that of pre-immune serum; (**B**) clonal reactivity of VHHs selected against CTX, CB and CA (brown). After three rounds of panning, 220 clones selected against crotoxin and CB and 88 against CA were subjected to ELISA assays. Of these, fifty eight (58), seventy eight (78) and two (02) were positive, respectively. All measurements were performed in triplicate. In white, gray and black are the positive clones in pannings 1, 2, and 3, respectively. The negative control (NC) was performed using pre-immune llama serum. Error bars represent standard deviation. An asterisk (*) indicates clones submitted to DNA sequencing; (**C**) amino acid sequence alignment of anti-crotoxin VHHs. Framework regions (FR), as well as complementarity determining regions (CDR) are indicated with arrows; two conserved cysteines are marked in red; VHH hallmark substitutions in FR2 are marked in blue, bolded and underlined. An additional cysteine pair, in clones KF498602 and KF498603, are shaded. The prominence of the CDR3 loop in clones KF498605 and KF498604, with 21 amino acid residues, is also worth noting. A colon (:) represents highly conserved amino acids; an asterisk (*) represents identical amino acid residues; a period (.) means somewhat similar but different amino acids and a blank represents dissimilar amino acids or gaps.

The *L. glama* immunization schedule, using complete and incomplete Freund’s adjuvant, proved to be satisfactory. An antiserum maximum titer (1 × 10^6^) was obtained after the second immunization, as observed by ELISA assays (Figure 1A). Following the final boost, cDNA was synthesized from total RNA extracted from 1.5 × 10^7^ lymphocytes. Amplification of the VHH gene repertoire by RT-PCR, and its recombination into the phagemid pHEN-1-6xHis allowed for VHH library construction with about 3.6 × 10^12^ clones using the *E. coli* TG1 strain. A total of 5.3 × 10^11^ recombinant phage particles were rescued after immune VHH library infection with the helper phage VCSM13. After three rounds of biopanning, carried out separately against CTX, CB and CA, 220 clones were selected for CTX, 220 for CB, and 88 for CA. All clones demonstrated the expected size for VHH by colony PCR. However, when tested by ELISA, 58 (26%), 76 (34%) and 2 (2%) VHHs showed absorbance values higher than the stipulated cut-off point (2 mean OD from negative samples plus two standard deviations), considered positive for CTX, CB, and CA, respectively (Figure 1B).

### 2.2. Sequence Analysis of Anti-Crotoxin VHHs

Ten clones, which showed the best absorbances in ELISA, were sequenced and a multiple sequence alignment between them revealed four sequence profiles for CB and one profile for CA. These sequences were deposited in the GenBank database under the following accession numbers: KF498602 (clone Ctx-17), KF498603 (clone Ctx-21), KF498604 (clone Cb-16), KF498605 (clone Cb-42) and KF498606 (clone Ca-12). All clones presented the established VHH hallmark amino acid in FR2 (Y/F37; E44; R45; G/F47), with the exception of KF498606. Besides that, the KF498606 showed a shorter CDR3 region with only five amino acid residues (FRGGVW) (Figure 1C). With 98% and 97% similarity, KF498602 and KF498603, and KF498604 and KF498605 presented CDR3 made of 18 (ATELISSCASTWYDAYSYW) and 21 (AASDEGTGSPGSLYTPDPYDY) amino acid residues, indicating that they originated from two common ancestral B cells. Although two conserved cysteines of VHH/VH, which form the canonical cross-species disulfide bond between FR1 and FR3, were observed in all the clones, only KF498602 and KF498603 presented an extra pair of cysteines, which may allow for the formation of an extra disulfide bond between FR2 and CDR3. After expressing recombinant pET22b-VHHs (KF498602, KF498603, KF498604, KF498605) in the *E. coli* strain BL21 (DE3), the clones were purified by IMAC. Approximately 2 to 6 mg of recombinant protein per liter of culture were obtained.

### 2.3. Cross-Immunoreactivity and Western Blot Analysis

**Figure 2:**
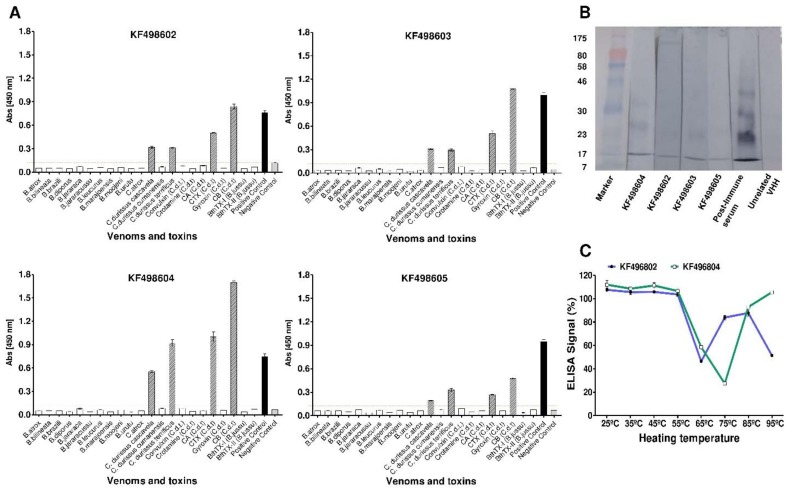
Evaluation of VHHs’ specificity and thermostability. (**A**) cross-reactivity of anti-crotoxin VHHs. *In vitro* reactivity showing different levels of interaction of selected VHHs KF498602; KF498604; KF498605; KF498606 with snake venoms and isolated toxins. Absorbance obtained by ELISA assays performed on plates coated with 10 µg/mL of each antigen and VHH at a ratio of 1:1 (*w*/*w*). The dashed lines represent the cut off. All measurements were performed in triplicate. For the negative control (NC), unrelated VHH was used in wells coated with CTX. Each value represents the mean ± SEM of triplicate samples; (**B**) specificity analysis of the VHHs with CB by Western blot. Immunoblotting demonstrating the interaction of the selected clones with monomeric (14 kDa) and multimeric forms of CB on a nitrocellulose membrane; (**C**) VHHs’ functional thermostability using ELISA. Signal percentage in ELISA against CB of KF496802 (blue) and KF496804 (green), after heating from 25 to 95 °C for 1 h, compared to the pre-heating samples. Each value represents the mean ± SEM of triplicate samples.

Besides being able to recognize CB, CTX and *C. durissus terrificus* venom, purified KF498602, KF498603, KF498604, and KF498605 clones were able to interact with *C. durissus cascavella* and *C. durissus collilineatus* venoms. However, when tested against toxins and venoms from the *Bothrops* genus, they showed no reactivity in ELISA, demonstrating their specificity for the *Crotalus* genus (Figure 2A). The clone KF498604 stood out for presenting the highest signal intensity, with an absorbance 10 times higher than the cut-off point. Clonal specificity was confirmed by Western blot analysis (Figure 2B), which demonstrated VHH binding capability for 14.2 kDa CB monomer forms, previously verified by a MALDI-TOF2 mass spectrometry system (AXIMA TOF2, Shimadzu, Japan) (data not shown). VHHs were also able to recognize multimeric forms of CB under non-reducing conditions by electrophoresis. The VHH KF498604 presented the greatest recognition capability, confirming the ELISA results.

### 2.4. Thermal Stability Evaluation of Selected VHHs and Interaction Analysis by SPR

When exposed to different temperature conditions for 1 h, KF498602 and KF498604 VHHs demonstrated reactivity against CB in ELISA. Both clones remained fully active in a temperature range from 25 to 55 °C, and above 75% when they were incubated at 85 °C. Between 55 and 85 °C, reduction in VHH activity was observed. However, KF498604 remained 100% active, even when heated to 95 °C (Figure 2C).

**Figure 3:**
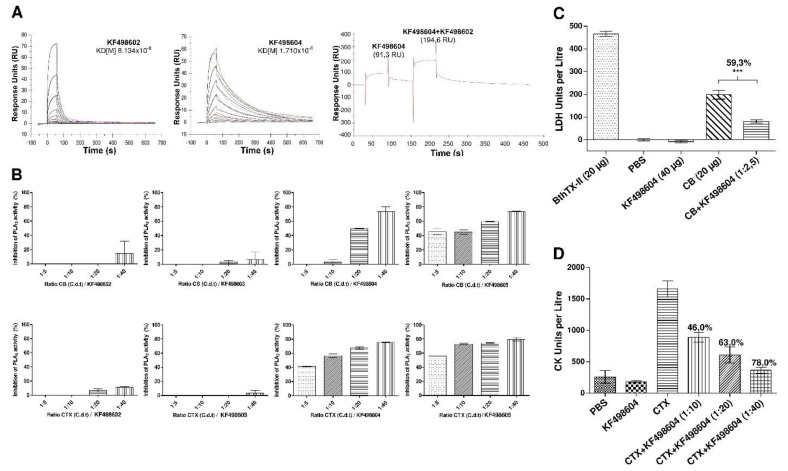
Evaluation of VHHs’ kinetic interaction and neutralization ability. (**A**) analysis of the kinetic interaction between VHHs and CTX by SPR. Sensorgrams obtained after injection of KF498604 and KF498602, at concentrations from 2.5 to 0.002 μM, on a crotoxin-coated CM5 chip. KD = affinity constant of the clones. Sensorgram obtained after injection of KF498604 and co-injection of KF498602. Signal gain (103 RU) on the sensorchip surface after KF498602 injection indicates that these clones interact with different epitopes of crotoxin; (**B**) *in vitro* inhibition of CB and CTX phospholipase activity by selected VHHs. Fluorimetric analysis of inhibition was assayed using synthetic fluorescent phospholipids and toxins pre-incubated with selected VHHs for 30 min at 37 °C in different proportions (1:5; 1:10 and 1:40 *w*/*w*). The toxin’s activity on the phospholipids, in the absence of VHH, was used as a positive control, and considered as having 100% activity. The negative controls were carried out using medium reactions with no toxins. Each value represents the mean ± SEM of duplicate samples; (**C**) inhibition of CB-induced cytotoxicity in murine C2C12 skeletal muscle myotubes by VHH. Cytotoxicity (cytolysis) was estimated by the release of lactic dehydrogenase (LDH) into the culture medium after 3 h of exposure to a pre-incubated solution containing CB and the KF498604 at a 1:2.5 (*w*/*w*) ratio. A significant reduction in LDL release was observed in cells exposed to the solution containing CB and VHH (59.3%, *** *p* < 0.05), when compared to samples incubated with CB alone; (**D**) *in vivo* neutralization of CTX-induced myotoxicity by VHH. Myotoxicity was estimated by plasma creatine kinase (CK) levels in Swiss mice after 3 h of intramuscular administration of the pre-incubated preparations (37 °C for 1 h) containing CTX and KF498604 VHH in proportions of 1:10, 1:20 and 1:40 (*w*/*w*). The negative control was performed with PBS or VHH, and as a positive control, animals were injected with CTX with no VHH addition. Each value represents the mean ± SEM. Bonferroni’s test was used to measure significance. A significant reduction in CK levels was observed in the plasma of mice exposed to CTX and VHH in all proportions (1:10 = 46%; 1:20 = 63%; 1:40 = 78%, *** *p* < 0.05) in relation to those exposed to the toxin alone.

The affinities of KF498602 and KF498604 VHHs for CTX were measured by SPR. After obtaining binding and dissociation sensograms, kinetic parameters, using the 1:1 Langmuir model, with highly reliable fit, made evident by the low Chi^2^ values obtained (≤1 RU), were determined (Table S1). While KF498604 showed affinity to CTX in a nanomolar range (KD = 81.3 nM), KF498602 demonstrated a lower affinity (KD = 1.7 µM). Injection of KF498202 at an equimolar rate on a CM5-CTX sensor chip, after saturation of the interaction signal between CTX and KF498604 (91.3 RU), promoted an increase of 113% in the signal gain (194.6 RU), suggesting that these clones interact with different antigenic determinants of the toxin (Figure 3A).

### 2.5. In Vitro and In Vivo Inhibition of CTX Activity by VHHs

The ability of the selected VHHs to inhibit the phospholipase activity of CTX and CB was evaluated by fluorimetry, using acyl-NBD-PC synthetic phospholipids. The results demonstrated the inhibitory potential of the VHHs KF498604 and KF498605 on the activity of the heterodimer CTX and its monomer CB. KF498604 was able to inhibit about 80% at the 1:40 (toxin/VHH) ratio, whereas KF498605 demonstrated inhibition of above 60% at a ratio of 1:20 (Figure 3B). The KF498602 and KF498603 clones showed no expressive toxin inhibition.

Neutralization of CB’s cytotoxic effect on murine myotubes by the KF498604 clone was verified using the pre-incubated solution at a ratio of 1:2.5 (toxin/VHH). The results demonstrated a significant reduction in released LDH levels (59.3%, *p* < 0.05), when compared to the control carried out with CB alone (Figure 3C). KF498604 VHH’s ability to inhibit the myotoxic effect of CTX, performed in vivo, was observed in all tested toxin/VHH ratios, especially at 1:40, which showed about 78.80% (*p* < 0.05) CK level reduction (Figure 3D).

### 2.6. Modelling and Interface Binding of the VHHs and CTX

**Figure 4:**
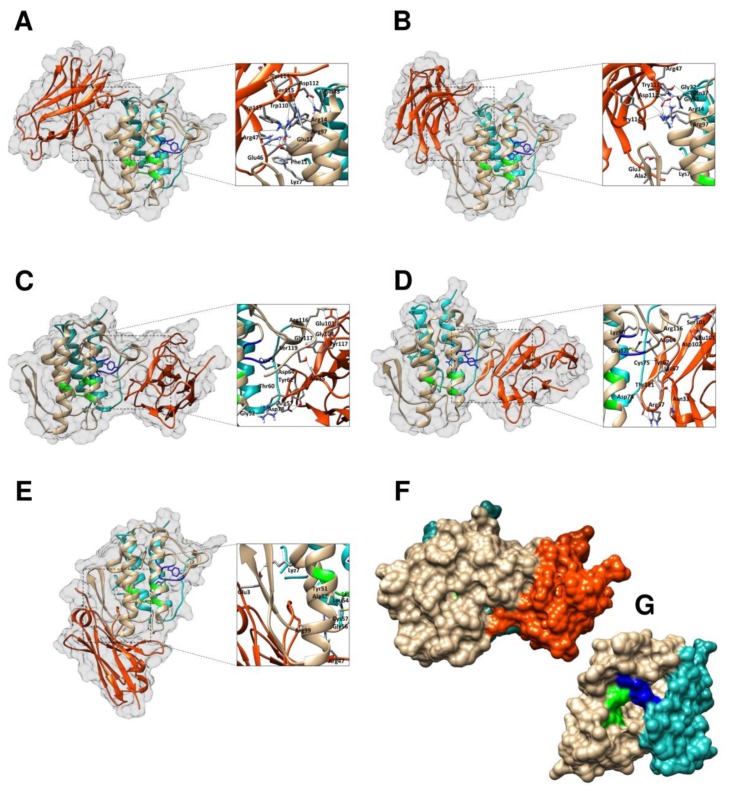
Docking results showing VHH binding sites on the surface of crotoxin, isoform CA2CBb. Cartoon representations of VHH-CTX interaction structures (side view) covered by a white surface, where α, β and γ chains of CA are shown in cyan ribbon, CB in light brown and the VHH is shown as an orange ribbon. The active site of CB (His48, Asp49, Tyr53 and Asp99) is represented in green and the Ca^2+^ binding loop (Try28, Cys29, Gly30, Trp31, Gly32) in blue. (**A**) KF498602-CTX; (**B**) KF498603-CTX; (**C**) KF498604-CTX; (**D**) KF498605-CTX; (**E**) KF498606-CTX; (**F**) surface representation of the KF498604–CTX interaction (side view); (**G**) surface representation of crotoxin’s crystal structure (front view, PDB ID 3R0L). Interaction sites were enlarged in order to show hydrogen bonds formed between amino acid residues. While the clones KF498602 and KF498603 recognize CB from a region opposite from the catalytic site (*N*-terminal α-helix A and α-helix D, mainly), KF498604 and KF498605 interact with the CA-CB interface of crotoxin, and, despite no contact with the catalytic site (His48, Asp49, Tyr53 and Asp99), sterically block the access of the substrate as demonstrated in (**C**,**D**,**F**). KF498605 VHH also interacts with CA in a region opposite from CB’s catalytic site.

Many putative templates of high-level similarity with the target sequences (KF498602, KF498603, KF498604, KF498605 and KF498606) were revealed through a BLAST search. The structures selected for modeling are shown in Table S2. The Ramachandran plot evaluation for selected VHHs showed more than 90% of amino acid residues in favorable regions. The model quality was also analyzed by comparing the predicted structure with the template via superimposition and atomic RMSD assessment. The RMSD of Cα trace between all homology structures and templates is less than 2.00 Å, indicating that the generated models are quite similar to the templates (Table S2). The best interactions between the VHHs and CTX are represented in Figure 4. Table 1 shows the hydrogen bonds formed between the VHHs and toxins in the final docking model.

## 3. Discussion

Antivenoms able to neutralize toxins in the blood and deep tissues and to present minimal adverse effects associated with the administration of non-human immunoglobulins are challenges the scientific community has faced over the years. Thus, different approaches have been proposed for innovative and cost-effective technologies to develop a new generation of antivenoms, given the global antivenom crisis [12,14,17,18,39].

Considering these challenges, crotalic envenoming’s epidemiology in Brazil, the relevance of CTX in envenoming by *C. durissus terrificus* snake venom, as well as the biotechnological versatility and therapeutic potential of camelid single domain antibodies, we selected anti-crotoxin VHHs following construction of an immunized *L. glama* VHH phage library.

*L. glama* immunization, performed following “the low-dose, low-volume, multi-site immunization protocol” [40], proved to be satisfactory. Animals presented antiserum maximum titer (1:10^6^), comparable with other immunization schedules [41,42]. With 3.6 × 10^12^ clones, the diversity of the immune VHH library was to be expected upon exploring antibody specificity and affinity developed naturally after immunization.

Three rounds of biopanning, performed in order to select high affinity clones [43], were enough to select four VHHs with anti-CB profiles (KF498602, KF498603, KF498604 and KF498605), and one VHH with an anti-CA profile (KF498606). VHH hallmark, presented in most profiles, are normally related to high hydrophilicity and remodeling capacity after exposure to denaturing temperatures (60–80 °C) [35,38,44]. An extra pair of cysteines in KF498602 and KF498603 may enable the formation of a disulfide bridge between FR2 and CDR3, which could confer greater rigidity to the CDR3 loop, increasing VHH stability [34,35,45]. Most clones showed longer CDR3 regions (18–21 amino acid residues) when compared to human VH (~14 aa). Indeed, VHH CDRs are usually larger to provide a compensatory antigenic surface given the absence of VL domains in HCAbs [34,46]. The low selection capability of anti-CA clones, the partial absence of VHH hallmark and the reduced CDR3 loop of KF498606 may be related to the absence of enzymatic or cytotoxic activity and, above all, to the immunomodulatory activity of this CTX subunit [47,48,49].

The four clones responsive to CB presented cross-reactivity only with snake venom from the genus *Crotalus*, thus demonstrating specificity, an important feature for snakebite diagnosis or serumtherapy [50,51]. Selected VHHs showed no interaction with other toxins (gyroxin, convulxin, crotamine) predominant in *C. durissus terrificus* venom [9]. Moreover, the CTX expression profile in *C. durissus cascavella*, *C. durissus collilineatus* and *C. durissus cumanensis* venoms is about 72.0%, 67.4% and 2.6%, respectively [52]. This variation justifies the VHHs’ differentiated reactivity between analyzed venoms. In addition, the anti-crotoxin VHHs were able to recognize monomeric and multimeric forms of CB in Western blot, after electrophoresis under non-reducing conditions [53], confirming ELISA results and clonal specificity.

In regards to thermal stability, the two analyzed VHH profiles demonstrated high resistance to heat-induced denaturation. Their stability against temperature changes, especially between 25 and 55 °C, is an important advantage for therapeutic and diagnostic applications, mainly in tropical and subtropical countries. It has been observed that VHH melting temperature values vary between 60 and 80 °C [44,54,55], justifying clone activity reduction at these temperatures. Although KF498602 presents an extra pair of cysteines in its amino acid sequence, suggested to form an extra dissulfide bond to stabilize VHH structure, this clone’s recognition capacity was reduced by 50% when heated to 95 °C. This was also observed in anti-triclocarban VHH, selected by [56]. The absence of this extra disulfide bond in KF498604 reinforces the idea that VHHs’ high thermostability is related to the typical amino acid substitutions present in camelid HCAbs [57]. Furthermore, both clones exhibited activity even after incubation at 85 °C (85%), while scFv [38], mAbs [58] and polyclonal IgG_1_ [56] are rendered inactive, demonstrating VHH robustness. Besides amino acid substitutions, the ability of VHH refold with high efficiency to its native conformation is probably due to the low complexity of VHH structure [57]. Performed selection allowed us to obtain clones with affinity in the micro and nanomolar range, as observed in previous studies, which select VHHs with a high capacity to recognize and neutralize animal toxins [41,42]. SPR analysis demonstrated that KF498604 showed better affinity to CTX (KD = 81.34 nM) than did KF498602 (KD = 1.7 μM), confirming results detected by ELISA and Western blot analyses. Moreover, SPR approaches were used to evaluate VHH interaction epitopes. Similar to that observed with anti-TcdA [59] and anti-hemagglutin VHHs [60], a signal gain of 103 RU on the sensorchip surface was observed with injection of the VHH KF498602 after saturation of the KF498604/CTX interaction signal. This suggests that these clones interact with different epitopes on the heterodimer.

KF498604 and KF498605 demonstrated significant inhibition of CTX and CB phospholipase activity in vitro. It has been observed that polyclonal and monoclonal antibodies, capable of inhibiting CB phospholipase activity in vitro, are potential inhibitors of CTX’s myotoxic and neurotoxic activity in vivo. This demonstrates the participation of CB in CTX’s toxic effects [61,62]. Because CTX does not present any effect on murine myotubes [63], CB was chosen to carry out cytotoxicity inhibition assays (Figure S1). The absence of CTX’s cytotoxic effect may be related to low dissociation of the heterodimer that is dependent on cell membrane sites, which may be absent or lower in the cellular models used [63,64]. VHH KF498604 was able to reduce LDH release related to the cytotoxic effect of CB under myotubes, demonstrating its ability to recognize and block CB̕s interaction with the cell membrane.

CK release, related to the myotoxic effect of CTX in mice, was significantly inhibited (*p* < 0.05) by the VHH KF498604 by up to 78% at the highest ratio (1:40 *w*/*w*), demonstrating this VHH’s potential protective effect *in vivo*. It was demonstrated that the myotoxic activity of the CB is related to its phospholipase activity, which is reduced by chemically modifying His48, an important residue of the catalytic site along with Asp99 [65]. KF498604 VHH was able to inhibit phospholipase activity as well as cytotoxic and myotoxic activity of CTX’s CB subunit, indicating some correlation between these effects.

The crystallographic structure of the CA2CBb isoform [10] demonstrates a partial blocking of substrate access to the CB subunit’s catalytic site through the interaction of residues Trp31 and Trp70 with Asp99 and Asp89 in the CA subunit. However, CB’s active site is still accessible from a lateral direction [66], which could explain the catalytic activity of the heterodimer CTX observed in assays with synthetic phospholipids and murine myotubes *in vitro*, as well as in in vivo myotoxicity evaluations.

Molecular docking suggested that KF498604 and KF498605 VHHs interact with the CA–CB interface of CTX, and, despite no contact with the catalytic site (His48, Asp49, Tyr53 and Asp99), the clones sterically block the substrate’s access (Figure 4F). Most contact surface occurs between VHHs and the CB subunit, justifying KF488604’s high inhibitory capacity against phospholipase, cytotoxic and myotoxic activities. Furthermore, both VHHs interact with residues of the CB’s C-terminal region, which could contribute to neurotoxic activity inhibition [67]. KF498602 and KF498603 recognize CB in a region opposite from the catalytic site (*N*-terminal α-helix A and α-helix D, mainly), which do not block substrate access [10]. As was previously proposed [66], CB’s *N*-terminal region, exposed to the solvent, may constitute an important pharmacological site of CTX. This region may allow CTX presynaptic acceptor binding in neuromuscular junctions, with subsequent dissociation of the CA subunit, enabling interaction of CB’s *C*-terminal with the target, and liberation of CB’s catalytic site, consequently triggering its neurotoxicity. Thus, although they do not inhibit phospholipase activity, these clones may be useful in studies on the neurotoxic mechanisms of CTX and neutralization assays.

Given the characteristics of the selected VHHs, as well as the involvement of systemic myotoxicity in Acute Kidney Injury (AKI), one of the main causes of death among victims of crotalic envenomation [68,69,70,71], we consider these clones promising tools for diagnosis or treatment of crotalic envenomation. Thus, these tools are being used in our laboratory as “modular blocks” to obtain different VHH products. Whether they be mono- or bispecific VHHs, or reconstituted HCAbs, the constructs will be great for generating humanized oligoclonal preparations against the major toxins in crotalic venoms. Protective capacity against neurotoxic and nephrotoxic effects triggered by CB, crotoxin, and crude venom is being investigated.

## 4. Materials and Methods

### 4.1. Ethics Statement

Procedures involving animals were performed in accordance with the recommendations of the National Council for the Control of Animal Experimentation (CONCEA), and were approved by the Ethics Commission on Animal Use (CEUA) of Fiocruz Rondônia under protocol numbers 2012/11 and 2014/11. The animals’ health status was checked routinely by a veterinarian. All animal groups (mice) were monitored regularly (5 min intervals) until the end of the experimental period and there was no unintended death of animals. After topical anesthesia (tetracaine ophthalmic drops) for retro-orbital bleeding, the mice were euthanized by cervical dislocation, according to CONCEA Normative Resolution No. 13, from 20 September 2013 (http://www.mct.gov.br/upd_blob/0228/228451.pdf). The licenses related to access to Brazilian genetic resources for scientific purposes are: Instituto Brasileiro do Meio Ambiente e dos Recursos Naturais Renováveis–IBAMA, Instituto Chico Mendes de Conservação da Biodiversidade–ICMBio (Number: 27131-1) and Conselho de Gestão do Patrimônio Genético–CGEN/Brazil (Number 010627/2011-1).

### 4.2. Camelid Immunization and Humoral Immune Response Monitoring

After evaluating the animals’ clinical status, one young *Lama glama* adult male was immunized subcutaneously five times, at weekly intervals, with increasing doses of CTX (100 µg and 200 µg), CB and CA subunits (50 µg and 100 µg each), plus complete or incomplete Freund’s adjuvant (Sigma-Aldrich, Saint Louis, MO, USA) (Table S3).

*L. glama*’s immune response was monitored by ELISA immunoassay. Microtiter plate wells (Nunc-MaxiSorp, Merck KGaA, Darmstadt, Germany) were coated with 1 µg of CTX, CB or CA diluted in PBS (NaCl 1.37 M, Na_2_HPO_4_·12H_2_O 8.5 × 10^−2^ M, KH_2_PO_4_ 1.5 × 10^−2^ M, KCl 2.7 × 10^−2^ M, pH 7.4) and incubated overnight at 4 °C. After being washed three times with PBST (0.05% Tween-20 in PBS), unspecific sites were blocked with PBSM (1% skimmed milk in PBS) for 24 h. Then, serial dilutions (1:10^2^; 1:10^3^; 1:10^4^; 1:10^5^, 1:10^6^) of the serum in PBSM, collected each week, were added to the wells and the plates were incubated for 24 h at 4 °C. The samples were washed with PBST, and rabbit anti-llama IgG_2_/IgG_3_ [72] at a 1:12,000 dilution in PBSM was added. Excess antibodies were removed by washing, and peroxidase conjugated mouse anti-rabbit IgG (Sigma Aldrich), at a 1:40,000 dilution in PBSM, was incubated for 5 h. The reaction was revealed using 100 µL/well of tetramethylbenzidine (TMB, Merck KGaA, Darmstadt, Germany), and blocked by 100 µL/well of sulfuric acid (0.32 M) after 30 min. Absorbances were measured at 450 nm in a microplate reader. The negative control was performed using llama pre-immune serum. All assays were carried out in triplicate.

### 4.3. Selection of Anti-Crotoxin VHHs 

Three days following the final boost, fifty milliliters of animal blood were collected to isolate lymphocytes using Ficoll-Paque Plus (Merck KGaA, Darmstadt, Germany). Total RNA was extracted from 1.5 × 10^7^ cells using Trizol Reagent (Thermo Fisher Scientific, Carlsbad, CA, USA), and cDNA synthesis was carried out with the SuperScript III First-Strand Synthesis System for RT-PCR (Invitrogen). A VHH gene repertoire, as well as an immune library, and recombinant phages expressing VHHs fused to VCSM13 helper phage protein III were obtained according to [38,42].

Selection of anti-crotoxin, anti-CB and anti-CA VHHs was performed by biopanning. To this, MaxiSorb immunotubes (Sigma-Aldrich) were adsorbed separately with 1 mg of CTX, CB, and CA in PBS and incubated for 24 h at 4 °C. Excess CTX, CB and CA were removed by washing with PBST and the samples were blocked in PBSM for 2 h. After being washed three times with PBST, recombinant VHH-phages, previously incubated in PBSM, were added to the immunotubes and the samples were incubated at 37 °C under homogenization for 30 min. Subsequently, samples were washed 15 times with PBST and 15 times with PBS. Bound VHH-phage particles were eluted with 100 mM HCl and neutralized with 1 M Tris-HCl, pH 7.5. The eluants were transferred separately to 10 mL of *E. coli* TG1 (A600 0.5) cultures and the samples were incubated for 1 h with no agitation and for 1 h with agitation at 37 °C. Following centrifugation at 4000× *g*, the supernatants were discarded, the pellets resuspended in 2YT, plated on 2YT/amp/glu, and incubated overnight at 30 °C. Individual clones were collected for two subsequent rounds of biopanning. Selected clones were analyzed by colony PCR and by ELISA in order to verify the presence and reactivity of VHHs against selected targets, respectively.

To perform the ELISA, positive mL of *E. coli* TG1 (A_600_ 0.5) cultures and the samples were incubated for 1 h with no agitation and for 1 h with agitation at 37 °C. Following centrifugation at 4000× *g*, the supernatants were discarded, the pellets resuspended in 2YT, plated on 2YT/amp/glu, and incubated overnight at 30 °C. Individual clones were collected for two subsequent rounds of biopanning. Selected clones were analyzed by colony PCR and by ELISA in order to verify the presence and reactivity of VHHs against selected targets, respectively. Clones were cultivated in 1 mL (2YT/amp), and VHH expression was induced with 3 mM isopropyl-d-thiogalactopyranoside (IPTG) (A_600_ 0.9) for 16 h at 30 °C. After centrifugation, 50 µL of supernatants containing soluble VHHs were used to carry out the assay, as described previously. Llama pre- and immune sera were used as negative and positive controls, respectively. Positive clones were sequenced, analyzed and submitted to GenBank.

### 4.4. Expression and Purification of Anti-Crotoxin VHHs

Four positive clones were subcloned into a pET-22b+ vector (Merck KGaA, Darmstadt, Germany). Thus, selected recombinant VHHs, cloned into pHEN-1-6xHis plasmid, were amplified by PCR using the following primers: VHNDEF (5′-GGAATTCCATATGGCCGA(G/C)GT(G/C)-′3) and VHXHOR (5′-CCGCTCGAGTGAGGAGACGG-′3). The restriction endonuclease recognition sites for *Nde*I and *Xho*I are underlined.

VHH fragments, composed of approximately 400 bp, were excised from agarose gel, digested with *Nde*I and *Xho*I endonucleases, and inserted into a pET-22b+ expression vector, in a frame with the polyhistidine (His) tag sequence. After transformation into *E. coli* BL21 (DE3) (New England Biolabs, Ipswich, MA, USA), the clones were grown in 30 mL of LB medium containing 100 μg/mL of ampicillin (LB amp), under agitation at 37 °C, overnight. Ten milliliters of these cultures were transferred to 1 L of LB amp and when its absorbance (A_600_) reached about 0.7, protein expression was induced with 1 mM IPTG. Cultures were incubated under agitation at 37 °C for 8 h. Following centrifugation, pellets were resuspended in 20 mL of 50 mM Tris-HCl (pH 8.0). In order to perform bacterial cell disruption, the samples were pretreated with 1 mg/mL of lysozyme at room temperature for 1 h and sonicated for 3.5 min, with 1 min pulses (Misonix Ultrasonic Processor, Qsonica, Newtown, CT, USA). Inclusion bodies were retrieved after centrifugation at 15,000× *g* for 15 min, resuspended in wash buffer (50 mM Tris-HCl, 50 mM Na_2_HPO_4_, 300 mM NaCl and 1% Triton X-100, pH 7.4) and incubated on ice for 10 min. This step was repeated once more. Triton X-100 was removed with Tris-HCl buffer. The samples were centrifuged and resuspended in dissolution buffer (50 mM Tris-HCl, 50 mM Na_2_HPO_4_, 300 mM NaCl and 8 M Urea), and then incubated at 4 °C for 1 h and centrifuged. VHHs were purified through immobilized metal affinity chromatography (IMAC) using Talon Co^2+^-precharged resin (GE Healthcare, Marlborough, MA, USA), under denaturing conditions, according to the manufacturer’s instructions. Renaturation of VHHs was performed by diafiltration in PBS, using 10 kDa Amicon Ultra-2 filter devices (Merck Millipore, Billerica, MA, USA). Soluble VHH protein concentration was determined by the Smith method (1985) [73] with a BCA protein kit (Thermo Fisher Scientific, Carlsbad, CA, USA).

### 4.5. Cross-Immunoreactivity Analysis of Anti-Crotoxin VHHs

In order to verify the specificity of the purified VHHs (KF498602, KF498603, KF498604 and KF498605), cross-immunoreactivity assays were performed by ELISA with different venoms and toxins from the *Bothrops* and *Crotalus* genera. Microtiter plate wells were coated with 1 µg of snake venoms obtained from Serpentário de Proteínas Bioativas, Batatais, Brazil (*B. atrox*, *B. bilineata*, *B. diporus*, *B. brasili*, *B. jararaca*, *B. jararacussu*, *B. leucurus*, *B. marajoensis*, *B. moojeni*, *B. urutu*, *C. atrox*, *C. durissus cascavella*, *C. durissus collilineatus*, *C. durissus cumanensis* and *C. durissus terrificus*), along with 1 µg of eight different toxins (Bothropstoxin I and II, from *B. jararacussu*, and convulxin, crotamin, crotapotin, crotoxin, and gyroxin from *C. durissus terrificus*). The plates were incubated at 4 °C overnight, washed with PBST and nonspecific sites were blocked with PBSM for 5 h. Subsequently, one microgram of the purified VHHs (pre-incubated in PBSM) was added and the assay was carried out as described previously. Llama immune serum (1:1000) was used as the positive control and an anti-BthTX VHH (KC329718) [42] (10 µg/mL), as the negative control. All assays were performed in triplicate.

### 4.6. Western Blot Analysis of Anti-Crotoxin VHHs

In order to confirm the specificity of VHHs for CB, Western blot analysis was performed. Ten micrograms of CB were reduced, electrophoresed on 12.5% SDS-PAGE, and transferred to a nitrocellulose membrane. Reactive sites were blocked with 5% skimmed milk in TBS buffer (TBSM) at 4 °C overnight. After being washed three times with TBS/0.1% Tween 20 (TBST), the strips were incubated with 0.2 mg/mL of each anti-crotoxin VHH (KF498602, KF498603, KF498604 and KF498605) overnight. The strips were washed again with TBST and incubated with rabbit anti-llama IgG2/IgG3 (1:1000 in 5% TBSM) overnight. After being washed, the samples were incubated with peroxidase conjugated mouse anti-rabbit IgG (1:3000 in 5% TBSM) for 6 h. The strips were washed, and the reactive signals were detected after incubation with hydrogen peroxide in diaminobenzidine (DAB) solution (SIGMAFAST™ DAB with Metal Enhancer Tablets, Sigma-Aldrich). Llama immune serum (1:1000) was used as the positive control and an anti-BthTX VHH (KC329718-0.2 mg/mL) [42], as the negative control.

### 4.7. Thermal Stability Analysis of Anti-Crotoxin VHHs

Thermal stability studies were performed in order to verify the binding capacity of selected anti-crotoxin VHHs in ELISA assays, after their exposure to different temperatures, according to Tabares-da Rosa et al. [56], with modifications. Forty microliters of purified KF498602 (0.440 μg/μL) and KF498604 (0.729 μg/μL) clones were incubated at 25, 45, 55, 65, 75, 85 and 95 °C in a thermal cycler (Applied Biosystems, Foster City, CA, USA) for 1 h. Samples were centrifuged at 2500× *g*, at room temperature for 5 min, and the supernatant concentrations were determined using the Smith method (1985) [73]. Soluble VHHs (1 µg) were incubated in PBSM and used to perform an ELISA assay in microtiter plates coated with 1 µg of CB, as described previously. The results were expressed as a signal percentage (450 nm) of the heated samples in relation to the control, corresponding to the unheated clones.

### 4.8. Interaction Analyses by Surface Plasmon Resonance (SPR)

The interaction analysis between KF498602 and KF4998604 clones and CTX was performed by SPR using a Biacore T200 system (GE Healthcare), according to [42], with modifications. The dextran layer of the CM5 sensor chip was activated by a 1:1 (*v*/*v*) mixture of 0.4 M EDC (1-Ethyl-3-3-dimethylaminopropyl carbodiimide) and 0.1 M NHS (*N*-hidroxysuccinimide) at a flow rate of 5 µL/min. CTX was diluted in C_2_H_3_NaO_2_ buffer (10 mM, pH 5.5) and injected into a selected flow cell for immobilization. A solution of 1 M H_2_NCH_2_CH_2_OH-HCl was injected in order to block the remaining reactive groups in the flow cell. The control flow cell was prepared with H_2_NCH_2_CH_2_OH–HCl alone. For kinetic measurements, CM5-CTX was subjected to serial dilutions of VHHs (2.5 to 0.002 µM) into the running buffer HBS-p (10 mM HEPES, 150 mM NaCl, 3.4 mM EDTA and 0.005% Tween 20, pH 7.4). The kinetic assay was performed at a flow rate of 30 µL/min at 25 °C. 

In order to verify whether or not the CTX interaction epitope is common for both clones, an additional assay was carried out. After saturation of KF498604 (2.5 μM)-CM5-CTX interaction, the sensor chip was washed with HBS-p running buffer, and KF498202 (1:1 VHH/VHH molar ratio) was injected. Following sensogram obtention, chip regeneration was performed with an AIW solution followed by an ICW solution for 30 s each (A-Equal volumes of C_2_H_2_O_4_, H_3_PO_4_, CH_2_O_2_, and ‎C_3_H_4_O_4_, each at 0.15 M, pH 5.0; C-20 mM EDTA; I-KSCN (0.46 M), MgCl_2_ (1.83 M), urea (0.92 M), guanidine-HCl (1.83 M); W-deionized water) according to Andersson; Hämäläinen and Malmqvist [74]. The binding responses were calculated by subtracting the RUs obtained from both blank control cells and running buffer injections. Kinetic analyses were performed by fitting the obtained sensograms with the 1:1 Langmuir model using BIA-evaluation software (version 1.0, GE Healthcare).

### 4.9. In Vitro Inhibition of CTX and CB Phospholipase Activities

Inhibition of CTX and CB phospholipase activities on fluorescent lipids were performed as described by [42]. The fluorescent phospholipid (Acyl-NBD-*acyl*-NBD-PC, Avanti Polar Lipids, Alabaster, AL, USA) was reconstituted in chloroform, dried over a low flow of N_2_, and dissolved in 150 mM NaCl to obtain a 1 mg/mL stock solution. Assays were performed in 150 µL in opaque plates, at wavelengths of 460 nm (excitation) and 534 nm (emission), 37 °C and at intervals of 3 s between each read, over 5 min, using Fluorescence Spectrometer Spectra Max M4 (Molecular Devices, San Jose, CA, USA) and SoftMax 6.0 Software (Molecular Devices, San Jose, CA, USA). The standard reaction medium contained 20 mM Tris–HCl (pH 7.5), 8 mM CaCl_2_ and 5 µM of phospholipids. In order to verify the VHHs’ ability to inhibit phospholipase activities, 0.08 µg and 0.04 µg of CTX and CB, respectively, were pre-incubated with the VHHs KF498202 and KF498604 (1:5; 1:10 and 1:40 *w*/*w*) for 30 min at 37 °C. The standard reactions with CTX or CB alone were used as positive controls. These reactions produced 186 and 115 fluorescence units, respectively, corresponding to 100% PLA_2_ activity. The negative control was performed with the standard reaction medium alone.

### 4.10. In Vitro Inhibition of CB Cytotoxicity by Selected VHHs

Inhibition of CB cytotoxicity by anti-crotoxin VHHs was evaluated by determining the lactic dehydrogenase (LDH) release from damaged murine myoblasts cells, differentiated in myotubes, as was previously described [64,75]. C2C12 cell culture was established in 25 cm^2^ bottles containing 5 mL of Dulbecco’s Modified Eagle’s Medium (DMEM, Sigma), supplemented with 10% fetal bovine serum (FBS, sigma), HEPES 2 g/L, HCO_3_^−^ 3.7 g/L, gentamicin (50 µg/mL) and incubated in a 5% CO_2_ atmosphere at 37 °C. After reaching full confluence, non-adherent cells were removed by washing with DMEM and 2 mL of 500 U/mL trypsin-EDTA solution (Sigma) was added. After incubation at 37 °C for 5 min, 5 mL of supplemented DMEM medium was added and the flasks were centrifuged at 300× *g*, 24 °C for 10 min. Cell pellets were resuspended in 1 mL of DMEM. An aliquot was diluted by 1:100 (*v*/*v*) in 0.4% trypan blue solution (Sigma) in order to count viable cells under an optical microscope (40×) using a Neubauer chamber. Cells were transferred to Costar^®^-type 96-well microplates (Sigma) at an initial density of 2.0 × 10^4^ cells/well and incubated under the same conditions until reaching 100% confluency. The supernatants were removed and 200 µL of DMEM supplemented with 1% FBS and gentamicin (50 µg/mL) was added for cell differentiation on myotubes [76]. Four to six days of incubation were required for the formation of a long layer of multinucleated myotubes, used in the cytotoxicity assays. For the neutralization assay, CB at its previously determined Minimal Cytotoxic Dose (20 µg) (Figure S1) was incubated at 37 °C for 1 h with the clone KF498604 at a ratio of 1:2.5 (*w*/*w*) in PBS. Then, 150 µL of the solution was transferred to wells containing the myotube culture in 50 μL of DMEM supplemented with 1% FBS. After incubation at 37 °C for 3 h, in a 5% CO_2_ atmosphere, supernatants were collected to determine LDH activity using diagnostic kits (Bioclin, Belo Horizonte, MG, Brazil). The results were expressed as LDH U/L. 0.1% Triton X-100 and 20 µg/well bothropstoxin II were used as positive controls and PBS as the negative control. All assays were performed in triplicate.

### 4.11. In Vivo Neutralization of CTX Myotoxicity by VHHs

The neutralizing effect on myotoxicity induced by CTX in mice was analyzed by determining the serum creatine kinase (CK) activity. Thus, CTX at its previously determined minimum myotoxic dose (MMD) (1.5 µg) (Figure S2) was incubated at 37 °C for 1 h with the VHH KF498604 at ratios of 1:10, 1:20 and 1:40 (*w*/*w*) in PBS. Then, groups containing 5 male mice (28–30 g) received an i.m. injection of 50 µL of toxin/VHH solutions in the gastrocnemius muscle. Positive control animals were injected with CTX and negative controls received 50 µL of PBS or VHH. After 3 h, blood was collected from the orbital plexus in order to determine the creatine kinase activity using diagnostic kits (Bioclin, Belo Horizonte, MG, Brazil). Results were expressed as CK U/L.

### 4.12. Modeling and Molecular Docking of Anti-Crotoxin VHHs

In order to investigate amino acid residues that participate in the antigen–antibody recognition, we performed homology modeling of five selected VHHs, following the methodology described by [42]. After modeling, the ClusPro2.0 server [77] was used to predict the interactions between modeled VHHs and the CTX isoform CA_2_CBb (PDB accession code: 3R0L). The antibody mode was selected with the non-CDR regions masked automatically [78]. The VHH structures were submitted as the receptor and CTX as the ligand. ClusPro selected the 1000 best scoring solutions, clustered them according to Root Mean Square Deviation (RMSD) considerations, and the lowest ClusPro score, representing the greatest probability of antigen-antibody interaction, was selected [79].

## Figures and Tables

**Table 1:** Molecular interactions between the amino acid residues of the VHHs and CTX.

Clone	VHH Domain	Amino Acids		CTX	Distances
		Antibody	Antigen	Domain	(Å)
KF498602	FR2	Arg-47	Glu-12	*N*-terminal α-helix A (CB)	1.8/1.9/1.8
	CDR3	Trp-117	Glu-12	*N*-terminal α-helix A (CB)	2
	FR2	Arg-47	Arg-14	*N*-terminal α-helix A (CB)	1.9
	FR2	Arg-47	Phe-11	*N*-terminal α-helix A (CB)	1.7
	CDR3	Ser-115	Arg-97	α-helix D (CB)	2.1
	CDR3	Tyr-114	Arg-97	α-helix D (CB)	1.8
	CDR3	Trp-110	Arg-14	*N*-terminal α-helix A (CB)	1.8
	FR2	Glu-46	Lys-7	*N*-terminal α-helix A (CB)	1.7
	CDR3	Asp-112	Gln-33	α-Chain (CA)	2.0/2.0
	FR2	Glu-46	Arg-14	*N*-terminal α-helix A (CB)	1.8
KF498603	CDR3	Asp-112	Arg-97	α-helix D (CB)	1.8/1.8/1.9
	CDR3	Asp-112	Arg-14	*N*-terminal α-helix A (CB)	1.9
	CDR3	Tyr-114	Arg-14	*N*-terminal α-helix A (CB)	1.7
	CDR3	Tyr-111	Gly-32	Ca^2+^ binding loop (CB)	1.8
	FR2	Arg-47	Gly-31	Ca^2+^ binding loop (CB)	1.7/2.2
	FR2	Arg-47	Gln-37	Loop (CB)	1.9
	FR1	Ala-2	Lys-z-7	*N*-terminal α-helix A (CB)	1.7
	FR1	Glu-3	Lys-7	*N*-terminal α-helix A (CB)	1.7
KF498604	FR3	Asp-64	Cys-75	β-wing (CB)	2.1
	CDR2	Arg57	Gly-52	α-helix C (CB)	1.7
	CDR3	Tyr-117	Gly117	*C*-terminal extension (CB)	1.8
	FR3	Try-62	Asp78	β-wing (CB)	1.9/2.5
	CDR2	Thr-60	Asp78	β-wing (CB)	2
	CDR3	Glu-103	Arg-116	*C*-terminal extension (CB)	2.0/1.9
	CDR3	Gly-104	Arg-116	*C*-terminal extension (CB)	1.7
	CDR1	Ala-35	Ser-119	*C*-terminal extension (CB)	2.2
KF498605	CDR2	Arg-57	Asp-78	β-Chain (CA)	1.8/1.8/2.0
	CDR1	Asn-33	Thr-121	*C*-terminal extension (CB)	1.8
	CDR3	Asp-102	Arg-116	*C*-terminal extension (CB)	1.9
	CDR3	Ser-101	Arg-116	*C*-terminal extension (CB)	2
	CDR3	Glu-103	Arg-116	*C*-terminal extension (CB)	1.2/1.8/1.8
	FR3	Asp-64	Lys-90	β-Chain (CA)	1.8
	FR3	Lyz67	Glu-87	β-Chain (CA)	1.7/1.8
	FR3	Tyr-62	Cys-75	β-Chain (CA)	2
KF498606	FR1	Glu-3	Lys-7	*N*-terminal α-helix A (CB)	1.7
	CDR3	Arg-99	Ala-52	α-Chain (CA)	1.9
	CDR3	Arg-99	Tyr-51	α-Chain (CA)	1.7/2.0
	CDR3	Arg-99	Cys-57	α-Chain (CA)	1.9/2.4
	CDR3	Arg-99	Leu-54	α-Chain (CA)	1.8
	FR2	Arg-47	Gly-56	α-Chain (CA)	1.9

## Supplementary Materials

The following are available online at http://www.mdpi.com/2072-6651/10/4/142/s1, Figure S1: Cytotoxic activity evaluation of Crotalus d. terrificus venom, CTX and CB in murine C2C12 skeletal muscle myoblasts; Figure S2: Determination of CTX myotoxic activity in mice; Table S1: Llama immunization schedule with CTX and CA and CB subunits; Table S2: Interaction analysis by SPR; Table S3: In silico studies’ data.

## References

[1] WHO A Systematic Technically Driven Process for the Adoption of Additional Diseases as NTDs. https://www.who.int/neglected_diseases/diseases/systematic_technically_driven_process/en/.

[2] Kasturiratne A., Wickremasinghe A.R., de Silva N., Gunawardena N.K., Pathmeswaran A., Premaratna R., Savioli L., Lalloo D.G., de Silva H.J. (2008). The global burden of snakebite: A literature analysis and modelling based on regional estimates of envenoming and deaths. PLoS Med..

[3] Chippaux J.P. (2015). Epidemiology of envenomations by terrestrial venomous animals in Brazil based on case reporting: From obvious facts to contingencies. J. Venom. Anim. Toxins Incl. Trop. Dis..

[4] Bochner R., Struchiner C.J. (2003). Snake bite epidemiology in the last 100 years in Brazil: A review. Cad. Saude Publica.

[5] Brasil. Ministério da Saúde. Secretaria de Vigilância em Saúde. Guia de Vigilância Epidemiológica. Caderno 14. Acidentes por Animais Peçonhentos. https://portal.saude.gov.br/portal/arquivos/pdf/gve_7ed_web_atual.pdf.

[6] Azevedo-marques M.M., Hering S.E., Cupo P., Cardoso J.L.C. (2009). Acidente Crotálico. Animais Peçonhentos no Brasil: Biologia, Clínica e Terapêutica dos Acidentes.

[7] Bucaretchi F., Herrera S.R.F., Hyslop S., Baracat E.C.E., Vieira R.J. (2002). Snakebites by *Crotalus durissus* ssp. in children in Campinas, São Paulo, Brazil. Rev. Inst. Med. Trop. São Paulo.

[8] Pinho F.M., Zanetta D.M., Burdmann E.A. (2005). Acute renal failure after *Crotalus durissus* snakebite: A prospective survey on 100 patients. Kidney Int..

[9] Georgieva D., Ohler M., Seifert J., von Bergen M., Arni R.K., Genov N., Betzel C. (2010). Snake Venomic of *Crotalus durissus terrificus* correlation with pharmacological activities. J. Proteome Res..

[10] Faure G., Xu H., Saul F.A. (2011). Crystal structure of crotoxin reveals key residues involved in the stability and toxicity of this potent heterodimeric β-neurotoxin. J. Mol. Biol..

[11] Theakston R.D., Warrell D.A., Griffiths E. (2003). Report of a WHO workshop on the standardization and control of antivenoms. Toxicon.

[12] Gutiérrez J.M., León G., Lomonte B. (2003). Pharmacokinetic-pharmacodynamic relationships of immunoglobulin therapy for envenomation. Clin. Pharmacokinet..

[13] De Silva H.A., Ryan N.M., de Silva H.J. (2016). Adverse reactions to snake antivenom, and their prevention and treatment. Br. J. Clin. Pharmacol..

[14] Brown N.I. (2012). Consequences of neglect: Analysis of the sub-saharan african snake antivenom market and the global context. PLoS Negl. Trop. Dis..

[15] Gutiérrez J.M. (2012). Improving antivenom availability and accessibility: Science, technology, and beyond. Toxicon.

[16] Morais V., Massaldi H. (2006). Economic evaluation of snake antivenom production in the public system. J. Venom. Anim. Toxins Incl. Trop. Dis..

[17] Alvarenga L.M., Zahid M., di Tommaso A., Juste M.O., Aubrey N., Billiald P., Muzard J. (2014). Engineering venom’s toxin-neutralizing antibody fragments and its therapeutic potential. Toxins.

[18] Laustsen A.H., Solà M., Jappe E.C., Oscoz S., Lauridsen L.P., Engmark M. (2016). Biotechnological trends in spider and scorpion antivenom development. Toxins.

[19] Huang Z., Phoolcharoen W., Lai H., Piensook K., Cardineau G., Zeitlin L., Whaley K.J., Arntzen C.J., Mason H.S., Chen Q. (2010). High-level rapid production of full-size monoclonal antibodies in plants by a single-vector DNA replicon system. Biotechnol. Bioeng..

[20] Siddiqui M.Z. (2010). Monoclonal Antibodies as Diagnostics; an Appraisal. Indian J. Pharm. Sci..

[21] Sharma M.C., Tuszynski G.P., Blackman M.R., Sharma M. (2016). Long-term efficacy and downstream mechanism of anti-annexinA2 monoclonal antibody (anti-ANX A2 mAb) in a pre-clinical model of aggressive human breast cancer. Cancer Lett..

[22] Chippaux J.P., Goyffon M. (1998). Venoms, antivenoms and immunotherapy. Toxicon.

[23] Tjandra J.J., Ramadi L., McKenzie I.F. (1990). Development of human anti-murine antibody (HAMA) response in patients. Immunol. Cell Biol..

[24] Nelson A.L. (2010). Antibody fragments: Hope and hype. MAbs.

[25] Roskos L.K., Davis C.G., Schwab G.M. (2004). The clinical pharmacology of therapeutic monoclonal antibodies. Drug Dev. Res..

[26] Batra S.K., Jain M., Wittel U.A., Chauhan S.C., Colcher D. (2002). Pharmacokinetics and biodistribution of genetically engineered antibodies. Curr. Opin. Biotechnol..

[27] Colcher D., Bird R., Roselli M., Hardman K.D., Johnson S., Pope S., Dodd S.W., Pantoliano M.W., Milenic D.E., Schlom J. (1990). In vivo tumor targeting of a recombinant single-chain antigen-binding protein. J. Natl. Cancer Inst..

[28] Nelson A.L., Reichert J.M. (2009). Development trends for therapeutic antibody fragments. Nat. Biotechnol..

[29] Pavlinkova G., Beresford G.W., Booth B.J., Batra S.K., Colcher D. (1999). Pharmacokinetics and biodistribution of engineered single-chain antibody constructs of MAb CC49 in colon carcinoma xenografts. J. Nucl. Med..

[30] Goel A., Colcher D., Baranowska-Kortylewicz J., Augustine S., Booth B.J., Pavlinkova G., Batra S.K. (2000). Genetically engineered tetravalent single-chain Fv of the pancarcinoma monoclonal antibody CC49: Improved biodistribution and potential for therapeutic application. Cancer Res..

[31] Miller B.R., Demarest S.J., Lugovskoy A., Huang F., Wu X., Snyder W.B., Croner L.J., Wang N., Amatucci A., Michaelson J.S. (2010). Stability engineering of scFvs for the development of bispecific and multivalent antibodies. Protein Eng. Des. Sel..

[32] Wang R., Xiang S., Feng Y., Srinivas S., Zhang Y., Lin M., Wang S. (2013). Engineering production of functional scFv antibody in *E. coli* by co-expressing the molecule chaperone Skp. Front. Cell. Infect. Microbiol..

[33] Hamers-Casterman C., Atarhouch T., Muyldermans S., Robinson G., Hamers C., Songa E.B., Bendahman N., Hamers R. (1993). Naturally occurring antibodies devoid of light chains. Lett. Nat..

[34] Muyldermans S. (2013). Nanobodies: Natural single-domain antibodies. Annu. Rev. Biochem..

[35] Kolkman J.A., Law D.A. (2010). Nanobodies—From llamas to therapeutic proteins. Drug Discov. Today Technol..

[36] Cortez-Retamozo V., Lauwereys M., Hassanzadeh G.G., Gobert M., Conrath K., Muyldermans S., De Baetselier P., Revets H. (2002). Efficient tumor targeting by single-domain antibody fragments of camels. Int. J. Cancer.

[37] Harmsen M.M., De Haard H.J. (2007). Properties, production, and applications of camelid single-domain antibody fragments. Appl. Microbiol. Biotechnol..

[38] Arbabi-Ghahroudi M., Desmyter A., Wyns L., Hamers R., Muyldermans S. (1997). Selection and identification of single domain antibody fragments from camel heavy-chain antibodies. FEBS Lett..

[39] Warrell D.A. (2012). Venomous Bites, Stings, and Poisoning. Infect. Dis. Clin. N. Am..

[40] Thanongsaksrikul J., Srimanote P., Maneewatch S., Choowongkomon K., Tapchaisri P., Makino S., Kurazono H., Chaicumpa W. (2010). A VHH that neutralizes the zinc metalloproteinase activity of botulinum neurotoxin type A. J. Biol. Chem..

[41] Richard G., Meyers A.J., McLean M.D., Arbabi-Ghahroudi M., MacKenzie R., Hall J.C. (2013). In vivo neutralization of a-cobratoxin with high-affinity Llama single domain antibodies (VHHs) and a VHH-Fc antibody. PLoS ONE.

[42] Prado N.D., Pereira S.S., da Silva M.P., Morais M.S., Kayano A.M., Moreira-Dill L.S., Luiz M.B., Zanchi F.B., Fuly A.L., Huacca M.E. (2016). Inhibition of the myotoxicity induced by *Bothrops jararacussu* venom and isolated phospholipases A2 by Specific Camelid SingleDomain Antibody Fragments. PLoS ONE.

[43] Bradbury A.R., Marks J.D. (2004). Antibodies from phage antibody libraries. J. Immunol. Methods.

[44] Dumoulin M., Conrath K., Van Meirhaeghe A., Meersman F., Heremans K., Frenken L.G., Muyldermans S., Wyns L., Matagne A. (2002). Single-domain antibody fragments with high conformational stability. Protein Sci..

[45] Govaert J., Pellis M., Deschacht N., Vincke C., Conrath K., Muyldermans S., Saerens D. (2012). Dual beneficial effect of interloop disulfide bond for single domain antibody fragments. J. Biol. Chem..

[46] Vu K.B., Arbabi-Ghahroudi M., Wyns L., Muyldermans S. (1997). Comparison of llama VH sequences from conventional and heavy chain antibodies. Mol. Immunol..

[47] Sampaio S.C., Hyslop S., Fontes M.R., Prado-Franceschi J., Zambelli V.O., Magro A.J., Brigatte P., Gutierrez V.P., Cury Y. (2010). Crotoxin: Novel activities for a classic beta-neurotoxin. Toxicon.

[48] Landucci E.C., Antunes E., Donato J.L., Faro R., Hyslop S., Marangoni S., Oliveira B., Cirino G., de Nucci G. (1995). Inhibition of carrageenin-induced rat paw oedema by crotapotin, a polypeptide complexed with phospholipase A_2_. Br. J. Pharmacol..

[49] Garcia F., Toyama M.H., Castro F.R., Proença P.L., Marangoni S., Santos L.M. (2003). Crotapotin induced modification of T lymphocyte proliferative response through interference with PGE_2_ synthesis. Toxicon.

[50] Janssen M., Freyvogel T.A., Meier J. (1990). Antigenic relationship between the venom of the nigth adder *Causus maculatus* and venoms of other viperids. Toxicon.

[51] Tan C.H., Tan N.H., Tan K.Y., Kwong K.O. (2015). Antivenom cross-neutralization of the venoms of *Hydrophis schistosus* and *Hydrophis curtus*, two common sea snakes in Malaysian waters. Toxins.

[52] Boldrini-França J., Corrêa-Netto C., Silva M.M., Rodrigues R.S., De La Torre P., Pérez A., Soares A.M., Zingali R.B., Nogueira R.A., Rodrigues V.M. (2010). Snake venomics and antivenomics of *Crotalus durissus* subspecies from Brazil: Assessment of geographic variation and its implication on snakebite management. J. Proteom..

[53] Marchi-Salvador D.P., Corrêa L.C., Magro A.J., Oliveira C.Z., Soares A.M., Fontes M.R. (2008). Insights into the role of oligomeric state on the biological activities of crotoxin: Crystal structure of a tetrameric phospholipase A2 formed by two isoforms of crotoxin B from *Crotalus durissus terrificus* venom. Proteins.

[54] Baral T.N., Murad Y., Nguyen T.D., Iqbal U., Zhang J. (2011). Isolation of functional single domain antibody by whole cell immunization: Implications for cancer treatment. J. Immunol. Methods.

[55] Olichon A., Schweizer D., Muyldermans S., de Marco A. (2007). Heating as a rapid purification method for recovering correctly-folded thermotolerant VH and VHH domains. BMC Biotechnol..

[56] Tabares-da Rosa S., Rossotti M., Carleiza C., Carrión F., Pritsch O., Ahn K.C., Last J.A., Hammock B.D., González-Sapienza G. (2011). Competitive selection from single domain antibody libraries allows isolation of high-affinity antihapten antibodies that are not favored in the llama immune response. Anal. Chem..

[57] Omidfar K., Rasaee M.J., Kashanian S., Paknejad M., Bathaie Z. (2007). Studies of thermostability in *Camelus bactrianus* (Bactrian camel) single-domain antibody specific for the mutant epidermal-growth-factor receptor expressed by *Pichia*. Biotechnol. Appl. Biochem..

[58] Van der Linden R.H., Frenken L.G., de Geus B., Harmsen M.M., Ruuls R.C., Stok W., de Ron L., Wilson S., Davis P., Verrips C.T. (1999). Comparison of physical chemical properties of llama VHH antibody fragments and mouse monoclonal antibodies. Biochim. Biophys. Acta.

[59] Hussack G., Arbabi-Ghahroudi M., van Faassen H., Songer J.G., Ng K.K., MacKenzie R., Tanha J. (2011). Neutralization of *Clostridium difficile* toxin A with single-domain antibodies targeting the cell receptor binding domain. J. Biol. Chem..

[60] Hufton S.E., Risley P., Ball C.R., Major D., Engelhardt O.G., Poole S. (2014). The breadth of cross sub-type neutralisation activity of a single domain antibody to influenza hemagglutinin can be increased by antibody valency. PLoS ONE.

[61] Choumet V., Jiang M.S., Radvanyi F., Ownby C., Bon C. (1989). Neutralization of lethal potency and inhibition of enzymatic activity of a phospholipase A_2_ neurotoxin, crotoxin, by non-precipitating antibodies (Fab). FEBS Lett..

[62] Oliveira J.G., Soares S.G., Soares A.M., Giglio J.R., Teixeira J.E., Barbosa J.E. (2009). Expression of human recombinant antibody fragments capable of partially inhibiting the phospholypase activity of *Crotalus durissus terrificus* venom. Basic Clin. Pharmacol. Toxicol..

[63] Ponce-Soto L.A., Lomonte B., Rodrigues-Simioni L., Novello J.C., Marangoni S. (2007). Biological and structural characterization of crotoxin and new isoform of crotoxin B PLA2 (F6a) from *Crotalus durissus collilineatus* snake venom. Protein J..

[64] Lomonte B., Angulo Y., Rufini S., Cho W., Giglio J.R., Ohno M., Daniele J.J., Geoghegan P., Gutiérrez J.M. (1999). Comparative study of the cytolytic activity of myotoxic phospholipases A2 on mouse endothelial (tEnd) and skeletal muscle (C2C12) cells in vitro. Toxicon.

[65] Soares A.M., Mancin A.C., Cecchini A.L., Arantes E.C., França S.C., Gutiérrez J.M., Giglio J.R. (2001). Effects of chemical modifications of crotoxin B, the phospholipase A2 subunit of crotoxin from *Crotalus durissus terrificus* snake venom, on its enzymatic and pharmacological activities. Int. J. Biochem. Cell Biol..

[66] Fernandes C.A., Pazin W.M., Dreyer T.R., Bicev R.N., Cavalcante W.L., Fortes-Dias C.L., Ito A.S., Oliveira C.L., Fernandez R.M., Fontes M.R. (2017). Biophysical studies suggest a new structural arrangement of crotoxin and provide insights into its toxic mechanism. Sci. Rep..

[67] Curin-Serbec V., Délot E., Faure G., Saliou B., Gubensek F., Bon C., Choumet V. (1994). Antipeptide antibodies directed to the c-terminal part of ammodytoxin a react with the PLA2 subunit of crotoxin and neutralize its pharmacological activity. Toxicon.

[68] Azevedo-Marques M.M., Cupo P., Coimbra T.M., Hering S.E., Rossi M.A., Laure C.J. (1985). Myonecrosis, myoglobinuria and acute renal failure induced by south american rattlesnake (*Crotalus durissus terrificus*) envenomation in Brazil. Toxicon.

[69] Gutiérrez J.M., Ponce-Soto L.A., Marangoni S., Lomonte B. (2008). Systemic and local myotoxicity induced by snake venom group II phospholipases A2: Comparison between crotoxin, crotoxin B and a Lys49 PLA2 homologue. Toxicon.

[70] Amaral C.F., de Rezende N.A., da Silva O.A., Ribeiro M.M., Magalhães R.A., dos Reis R.J., Carneiro J.G., Castro J.R. (1986). Insuficiência renal aguda secundária a acidentes ofídicos botrópico e crotálico. Análise de 63 casos. Rev. Inst. Med. Trop. Sao Paulo.

[71] Jorge M.T., Ribeiro L.A. (1992). The epidemiology and clinical picture of an accidental bite by the South American rattlesnake (*Crotalus durissus*). Rev. Inst. Med. Trop. Sao Paulo.

[72] Pereira S.S., Moreira-Dill L.S., Morais M.S., Prado N.D., Barros M.L., Koishi A.C., Mazarrotto G.A., Gonçalves G.M., Zuliani J.P., Calderon L.A. (2014). Novel camelid antibody fragments targeting recombinant nucleoprotein of Araucaria hantavirus: A prototype for an early diagnosis of hantavirus pulmonary syndrome. PLoS ONE.

[73] Smith P.K., Krohn R.I., Hermanson G.T., Mallia A.K., Gartner F.H., Provenzano M.D., Fujimoto E.K., Goeke N.M., Olson B.J., Klenk D.C. (1985). Measurement of protein using bicinchoninic acid. Anal. Biochem..

[74] Andersson K., Hämäläinen M., Malmqvist M. (1999). Identification and optimization of regeneration conditions for affinity- based biosensor assays. A multivariate cocktail approach. Anal. Chem..

[75] Lomonte B., Tarkowski A., Bagge U., Hanson L.A. (1994). Neutralization of the cytolytic and myotoxic activities of phospholipases A2 from *Bothrops asper* snake venom by glycosaminoglycans of the heparina/heparan sulfate family. Biochem. Pharmacol..

[76] Ebisui C., Tsujinaka T., Morimoto T., Kan K., Iijima S., Yano M., Kominami E., Tanaka K., Monden M. (1995). Interleukin-6 induces proteolysis by activating intracellular proteases (cathepsins B and L, proteasome) in C2C12 myotubes. Clin. Sci. (Lond.).

[77] Chen R., Li L., Weng Z. (2003). Zdock: An initial-stage protein docking algorithm. Proteins.

[78] Brenke R., Hall D.R., Chuang G.Y., Comeau S.R., Bohnuud T., Beglov D., Schueler-Furman O., Vajda S., Kozakov D. (2003). Application of asymmetric statistical potentials to antibody-protein docking. Bioinformatics.

[79] Kozakov D., Brenke R., Comeau S.R., Vajda S. (2006). PIPER: An FFT-based protein pocking program with pairwise potentials. Proteins.

