# Supplementary material for: Camelid Single-Domain Antibodies (VHHs) against Crotoxin: A Basis for Developing Modular Building Blocks for the Enhancement of Treatment or Diagnosis of Crotalic Envenoming
Source: Toxins (Basel). 2018 Mar 29;10(4):142. doi: 10.3390/toxins10040142 (PMC5923308; doi:10.3390/toxins10040142)
Supplement: Supplementary file 1 [file toxins-10-00142-s001.pdf]

Supplementary Materials: Camelid Single-Domain Antibodies (VHHs) against Crotoxin: A Basis for Developing Modular Building Blocks for the Enhancement of Treatment or Diagnosis of Crotalic Envenoming

Marcos B. Luiz [1,2], Soraya S. Pereira [1], Nidiane D. R. Prado [1], Naan R. Gonçalves [1], Anderson M. Kayano [1], Leandro S. Moreira-Dill [1], Juliana C. Sobrinho [1], Fernando B. Zanchi [1], André L. Fuly [3], Cleberson F. Fernandes [4], Juliana P. Zuliani [1,5], Andreimar M. Soares [1,6,*], Rodrigo G. Stabeli [7,8] and Carla F. C. Fernandes [1,*]

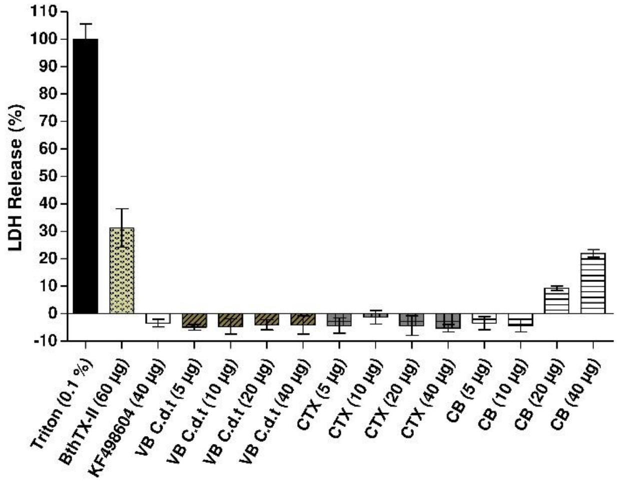

Figure S1. Cytotoxic activity evaluation of *Crotalus d. terrificus* venom, CTX and CB in murine C2C12 skeletal muscle myoblasts.

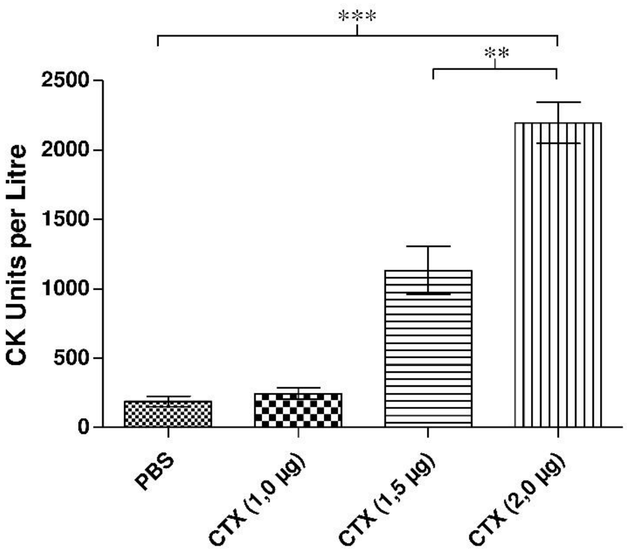

Figure S2. Determination of CTX myotoxic activity in mice.

Table S1. Interaction analysis by SPR.

Immobilized antigen	VHH	k_{on} [1/Ms]	k_{off} [1/s]	R_{max}[RU]	K_D [M]	Chi^2 [RU]
CTX	KF498602	$2.148x10^{+4}$	0.03673	67.93	$1.710x10^{-6}$	0.764
	KF498204	$6.807x10^{+4}$	0.005537	47.18	$8.134x10^{-8}$	1.10

Binding kinetics parameters were measured according to SPR sensorgrams shown in Figure 3 using the 1:1 Langmuir model

Table S2. In silico studies' data

Target (VHH clone)	Template (PDB:chain)	% Identity	Resolution (Å)	RMSD (Å)	Reference Template
KF498602	1xfp:A	64	1.50	1.95	[1]
KF498603	1xfp:A	64	1.50	0.21	[1]
KF498604	1kxt:B	66	2.00	0.36	[2]
KF498605	2vxs:K	76	2.63	0.29	[3]
KF498606	4b41:A	78	1.19	0.27	[4]

Table S3. Llama immunization schedule with CTX and CA and CB subunits.

Day	Immunization number	Toxin dose			Adjuvant	Serum collection
		CTX	CA	CB		
-7	-	-	-	-	-	Yes
0	1	100 µg	50 µg	50 µg	-	Yes
7	2	100 µg	50 µg	50 µg	50 µL CFA[a]	Yes
14	3	200 µg	100 µg	100 µg	100 µL IFA[b]	Yes
21	4	200 µg	100 µg	100 µg	100 µL IFA	Yes
28	5	200 µg	100 µg	100 µg	100 µL IFA	Yes
31	-	-	-	-	-	Yes

[a]CFA: Complete Freund's adjuvant; [b]IFA: Incomplete Freund's adjuvant; The llama was immunized with CTX, CA and CB subunits by subcutaneous route. Blood was collected from the jugular vein three days after final boost.

References

1. De Genst, E.; Handelberg, F.; Van Meirhaeghe, A.; Vynck, S.; Loris, R.; Wyns, L.; Muyldermans, S. Chemical basis for the affinity maturation of a Camel Single Domain Antibody. *J. Biol. Chem.* **2004**, *279*, 53593–53601.
2. Desmyter, A.; Spinelli, S.; Payan, F.; Lauwereys, M.; Wyns, L.; Muyldermans, S.; Cambillau, C. Three camelid VHH domains in complex with porcine pancreatic alpha-amylase. Inhibition and versatility of binding topology. *J. Biol. Chem.* **2002**, *277*, 23645–23650.
3. Gerhardt, S.; Abbott, W.M.; Hargreaves, D.; Pauptit, R.A.; Davies, R.A.; Needham, M.R.; Langham, C.; Barker, W.; Aziz, A.; Snow, M.J.; et al. Structure of IL-17A in complex with a potent, fully human neutralising antibody. *J. Mol. Biol.* **2009**, *394*, 905–921.
4. Beringer, D.X.; Dorresteijn, B.; Rutten, L.; Wienk, H.; El Khattabi, M.; Kroon-Batenburg, L.M.J.; Verrips, C.T. Crystal structure of an amyloid-beta binding Single Chain Antibody G7 Protein Data Bank. *Code 4b41* 2012. Available online: https://www.rcsb.org/pdb/explore.do?structureId=4B41 (accessed on 15 December 2017).

© 2018 by the authors. Submitted for possible open access publication under the terms and conditions of the Creative Commons Attribution (CC BY) license (http://creativecommons.org/licenses/by/4.0/).
